# Preoperative frailty parameters as predictors for outcomes after transcatheter aortic valve implantation: a systematic review and meta-analysis

**DOI:** 10.1007/s12471-020-01379-0

**Published:** 2020-03-18

**Authors:** M. S. van Mourik, J. F. Velu, V. R. Lanting, J. Limpens, B. J. Bouma, J. J. Piek, J. Baan, J. P. S. Henriques, M. M. Vis

**Affiliations:** 1 Department of Cardiology, Amsterdam UMC, University of Amsterdam, Amsterdam Cardiovascular Sciences, Amsterdam, The Netherlands; 2 Medical Library, Amsterdam UMC, University of Amsterdam, Amsterdam, The Netherlands

**Keywords:** Transcatheter aortic valve implantation, Frailty, Predictor, Outcome, Mortality

## Abstract

Guidelines suggest using frailty characteristics in the work-up for a transcatheter aortic valve implantation (TAVI). There are many frailty-screening tools with different components. The prognostic value of the individual parameters in frailty is as yet unclear. The objective of this systematic review and meta-analysis was to find and pool predictors for 1‑year mortality after TAVI. We followed a two-step approach. First, we searched for randomised controlled trials on TAVI to identify frailty parameters used in these studies. Second, we searched for publications on these frailty parameters. Articles were included for pooled analysis if the studied frailty parameters were dichotomised with clear cut-off values based on common standards or clinical practice and reported adjusted hazard ratios (HR) of 1‑year mortality after TAVI. We calculated pooled effect estimates of 49 studies based on dichotomised frailty scores (HR: 2.16, 95% CI: 1.57–3.00), chronic lung disease (HR: 1.57, 95% CI: 1.45–1.70), estimated glomerular filtration rate <30 ml/min (HR: 1.95, 95% CI: 1.68–2.29), body mass index <20 kg/m^2^ (HR: 1.49, 95% CI: 1.09–2.03), hypoalbuminaemia (HR: 1.77, 95% CI: 1.38–2.25), anaemia (HR: 2.08, 95% CI: 0.93–4.66), low gait speed (HR: 13.33, 95% CI: 1.75–101.49) and Katz activities of daily living (ADL) score of 1 or more deficits (HR: 5.16, 95% CI: 0.77–34.47). Chronic lung disease, chronic kidney disease, underweight, hypoalbuminaemia, a low frailty score, anaemia, low gait speed and an ADL deficiency were associated with worse 1‑year outcomes after TAVI.

**Electronic supplementary material:**

The online version of this article (10.1007/s12471-020-01379-0) contains supplementary material, which is available to authorized users.

## Introduction

Aortic valve stenosis is the most prevalent valvular heart disease in the Western population and is associated with ageing. Transcatheter aortic valve implantation (TAVI) has evolved as a routine treatment for patients with severe aortic valve stenosis at high or prohibitive risk for surgical aortic valve replacement [1, 2]. TAVI-specific risk stratification models are currently lacking as surgical risk models poorly predict TAVI outcomes [3, 4].

Guidelines suggest using frailty characteristics in the selection of patients for TAVI [1, 2]. Frailty is associated with diminished outcomes and increased risk of mortality and morbidity after surgical procedures. The Valve Academic Research Consortium uses the multifactorial definition of slowness, weakness, wasting and malnutrition, poor endurance, inactivity and limitation of independence [5]. There are numerous methods to assess frailty, although a standard and objective measurement method is lacking [6], and most consist of the quantification or dichotomisation of comorbidities. According to the Fried criteria, a patient is frail if three of the following criteria are met: a decline in lean body mass, strength, endurance, balance, walking performance and low physical activity [7]. Rockwood et al. defined frailty as an accumulation of deficits, and frailty is therefore quantified as the number of deficits a patient expresses as opposed to the nature of the health problems [8]. Frailty can also be described as the absence of resilience to react to (external) stressors such as a medical procedure or development of illness, but frailty and resilience are often used interchangeably [9].

The prognostic value of the different components of frailty in TAVI patients is as yet unclear.

The objective of this systematic review and meta-analysis was to find and pool frailty characteristics as predictors for 1‑year mortality after TAVI.

## Methods

### Search strategy

We followed the PRISMA (Preferred Reporting Items for Systematic Reviews and Meta-Analyses) guidelines for reporting. The review was conducted in a two-step approach. First Ovid MEDLINE was searched to find randomised clinical trials on TAVI (last update: 12 April 2018) (Electronic Supplementary Material; search strategy, point 6). The full text of relevant randomised controlled trials (RCTs) was read to identify frailty parameters used in the studies. A more extensive list of the frailty variables found can be seen in the Electronic Supplementary Material (frailty characteristics search 1 RCT). We grouped variables together if they were similar or reflected the reciprocal definition such as body mass index (BMI), overweight and underweight.

Second, a search was performed in Ovid MEDLINE and Ovid Embase from inception to 12 April 2018 for TAVI and frailty (in general) or specific frailty parameters, previously identified in the RCTs (step 1). All searches were designed and performed by an experienced information specialist (J.L.), using both controlled terms (i.e. MeSH terms) and text words. There were no date, language or other restrictions, except for Embase, where we excluded MEDLINE records, editorials and conference abstracts. The records retrieved were imported and deduplicated in EndNote X7.5. The cited and citing references of the included studies were screened for additional relevant publications.

The complete search strategies can be found in the Electronic Supplementary Material (complete search).

### Eligibility criteria (and study selection)

We included peer-reviewed, English-language original studies in humans. There were no restrictions as regards study participants, vascular access route, percutaneous valve type or other TAVI procedural characteristics. Primary outcome was all-cause 1‑year mortality. Available data with described hazard ratios (HRs) for the primary outcome were included.

### Data extraction

Two reviewers (M.S.v.M. and J.F.V.) independently screened title, abstracts and full texts of the identified publications using Rayyan [10]. Disagreements were solved by consensus or a third reviewer (M.M.V.). For each study included in the analysis, TAVI population demographics (age, gender, STS score and EuroSCORE), outcome descriptions (HR and 95% confidence interval (CI) or other effect estimates) and length of follow-up were collected. Articles were finally included for pooled analysis if the described predictor was dichotomised with clear cut-off values based on common standards or clinical practice and reported adjusted HRs. If the same study was reported twice, only the earliest published results were used in the pooled analysis.

We analysed various reported frailty scores (dichotomised as frail/non-frail according to the scoring system used), any described chronic lung disease (CLD), kidney disease (estimated glomerular filtration rate (eGFR) <30 ml/min or creatinine >200 µmol/l), underweight (BMI <20 kg/m^2^), hypoalbuminaemia (<3.5 g/dl or <4 g/dl), independence (Katz activities of daily living (ADL) score of 1 or more deficits), gait speed (<6 s on 5‑m walking test) and anaemia (cut-off for males: <13 g/dl, females: <12 g/dl).

### Assessment of study quality and risk of bias

Studies were first assessed for quality by two reviewers and marked as ‘high’ or ‘low’ quality, based on full-text review of the methodology and reporting. A structured assessment of study quality was done using the Newcastle-Ottawa scale, in which sample selection, comparability and outcomes were scored (Electronic Supplementary Material, frailty characteristics search 1 RCT).

### Statistical analysis

Articles were divided into subthemes based on the parameters found in the initial search for frailty and comorbidities with a described relation to frailty. Given the fact that there was wide variance between study designs and analysis, forest plots for random effects models were created to calculate summary effect estimates within the subthemes. Heterogeneity was assessed using the Cochran’s Q test for heterogeneity. Statistical analysis was performed in R (version 3.3.3, http://www.r-project.org, R Foundation for Statistical Computing, Vienna, Austria) and the packages ‘metafor’ [11] and ‘meta’ [12]. Categorical variables are presented as numbers with percentages, means and standard deviations (SDs) or medians and interquartile ranges (IQRs) as appropriate. A *p*-value of <0.05 was considered statistically significant.

## Results

**Fig. 1:**
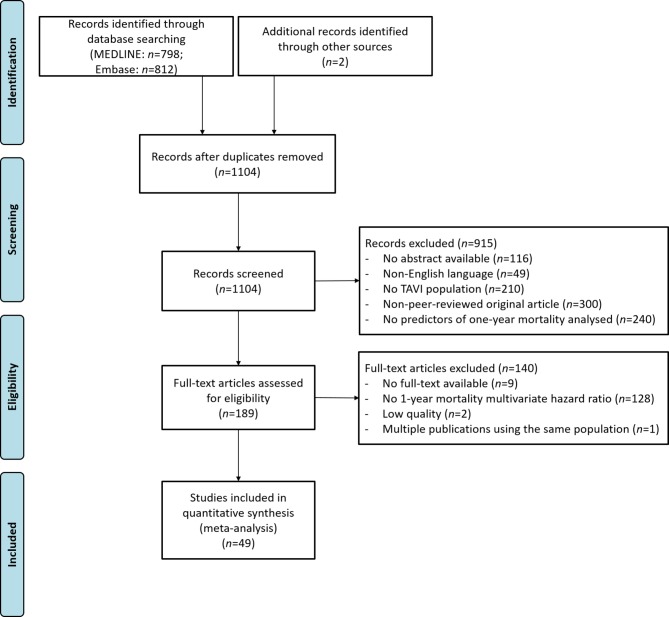
Flowchart. *TAVI* transcatheter aortic valve implantation, *HR* hazard ratio

The first literature search identified 315 putative RCTs on TAVI articles. Multiple frailty parameters were found and categorised (Electronic Supplementary Material, frailty characteristics search 1 RCT). The second search (TAVI plus frailty parameters, identified by search 1) identified 1104 records, of which 49 articles were included in the meta-analysis and are presented in the PRISMA flow diagram (Fig. 1).

The main characteristics and findings of the studies included are summarised in Tab. 1. Some studies reported both on univariate and multivariate results of HR of the frailty characteristic for 1‑year mortality. An overview and comparison of univariate and multivariate HR per variable can be found in the Electronic Supplementary Material (frailty characteristics search 1 RCT). There were no significant differences in pooled HRs between univariate and multivariate analysis.

**Table 1:** Baseline characteristics of studies included in the analysis per frailty parameter, in order of the cumulative number of patients included

Frailty parameter	Chronic lung disease	eGFR 30 ml/min	BMI <20 kg/m^2^	Hypoalbuminaemia	Frailty	Anaemia	Gait speed	ADL independence
Number of studies	27	8	7	5	9	5	3	2
Cumulative number of patients	36,484	9993	9251	6347	5876	3746	1501	478
Number of patients (median, IQR)	476 (350, 1218)	802 (508, 1181)	1215 (529, 1756)	1215 (150, 1215)	498 (339, 734)	549 (182, 1201)	148 (125, 702)	239 (194, 285)
Age (years), mean (SD)	82.1 (1.6)	81.8 (1.3)	83.2 (1.2)	83.6 (1.6)	82.2 (1.2)	80.2 (3.0)	81.5 (0.7)	82.8 (1.1)
Male gender (%), mean (SD)	47.8 (9.4)	49.3 (6.4)	44.9 (15.0)	35.1 (12.1)	48.0 (6.6)	51.4 (2.4)	41.8 (15.6)	40.6 (4.0)
EuroSCORE I (%), mean (SD)	20.3 (3.21)	20.32 (2.24)	21.02 (2.94)	17.53 (3.36)	16.90 (3.58)	15.30 (3.19)	15.37 (1.95)	16.85 (3.32)
EuroSCORE II (%), mean (SD)	7.63 (1.56)	7.91 (1.71)	7.30 (NA)	5.16 (0.79)	7.97 (3.35)	4.00 (NA)	5.10 (NA)	NaN (NA)
STS (%), mean (SD)	8.78 (2.88)	10.01 (3.57)	8.21 (1.40)	6.80 (1.31)	8.24 (1.79)	5.80 (0.28)	8.70 (2.98)	6.10 (0.14)

*eGFR* estimated glomerular filtration rate, *BMI* body mass index, *ADL* activities of daily living, *IQR* interquartile range, *SD* standard deviation, *STS* Society of Thoracic Surgery score

Studies reported on specific frailty scores as well as comorbidities/conditions which are related to frailty. Articles which reported on frailty assessment as well as comorbidities were taken into the analysis for each subtheme.

### Chronic lung disease

**Fig. 2:**
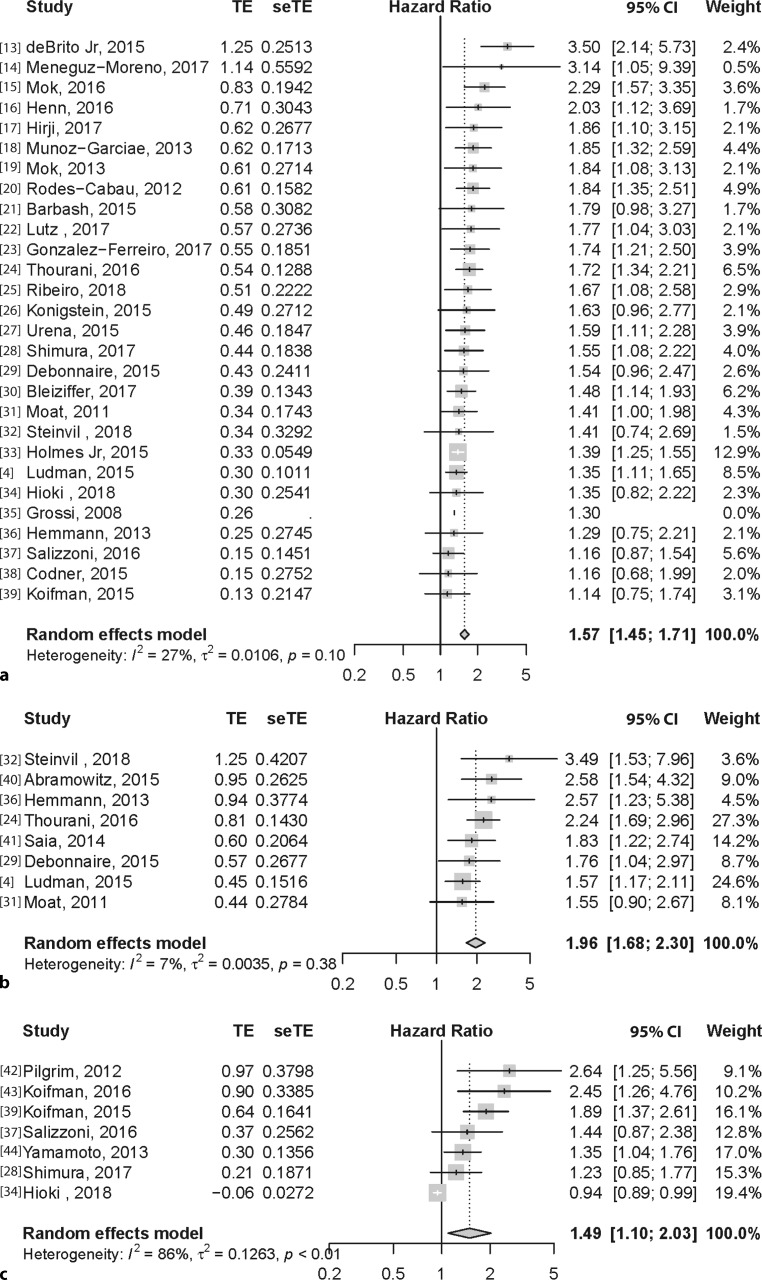
**a**–**h** Forest plot per variable (multivariate HR for 1‑year mortality after transcatheter aortic valve implantation). **a** Chronic lung disease. **b** Estimated glomerular filtration rate <30 ml/min. **c** Body mass index <20. **d** Hypoalbuminaemia. **e** Low frailty score. **f** Anaemia. **g** Low gait speed. **h** Deficiency in activities of daily life. *HR* hazard ratio, *TE* treatment effect, *seTE* standard error treatment effect

Twenty-seven studies [4, 13–39] (36,484 patients) were included in the analysis for the effect of CLD. One study was not used in the pooled analysis because the article did not report confidence intervals of the HR [35]. The pooled result of the random effects model was an HR 1.57 (95% CI: 1.45–1.71), as shown in Fig. 2a. Heterogeneity analysis showed that there was no significant difference in the between-study variance (*I*^2^: 27%, τ = 0.0106, *p* < 0.10) (Fig. 2a).

### Chronic kidney disease (eGFR <30 ml/min)

Eight studies [4, 24, 29, 31, 32, 36, 40, 41] (9993 patients) were included in the analysis for patients with an eGFR below 30 ml/min as surrogate for chronic kidney disease (CKD). The pooled result was an HR of 1.96 (95% CI: 1.68–2.30). Heterogeneity analysis showed that there was no significant difference in the between-study variance (*I*^2^ = 7%*, *τ^2^ = 0.0035,* p* = 0.38) (Fig. 2b).

### Underweight (BMI <20)

Seven studies [28, 34, 37, 39, 42–44] (9251 patients) were included in the analysis for patients who were underweight (BMI <20 kg/m^2^) at baseline. The pooled result of the random effects model was an HR of 1.49 (95% CI: 1.10–2.03). There was a significant difference in the between-study variance (*I*^2^ = 86%, τ^2^ = 0.1263, *p* < 0.01) (Fig. 2c).

### Hypoalbuminaemia

Five studies [28, 45–48] (6347 patients) were included in the analysis regarding hypoalbuminaemia at baseline and resulted in a pooled HR of 1.77 (95% CI: 1.38–2.26). Heterogeneity analysis showed that there was no difference in between-study variance (*I*^2^ = 28%, τ^2^ = 0.0214, *p* = 0.24) (Fig. 2d).

### Frail according to frailty score

Nine studies [20, 32, 38, 40, 49–52] (5876 patients) were included in the analysis for patients who were frail according to a frailty score. Different frailty scores were used among the studies such as the Canadian Study of Health and Aging Scale [49]; the PARTNER frailty definition (a composite of different markers: serum albumin, dominant hand grip strength, gait speed, Katz ADL survey) [40, 50]; a cut-off on failing on three or more categories of the aforementioned PARTNER frailty markers, with the addition of BMI <20 kg/m^2^ [32]; at least three of five criteria: muscle weakness, slow gait speed, low physical activity, exhaustion and unintentional weight loss [51]; Rockwood scale [52]; Clinical Frailty scale or a physician perceived frailty status based on clinical status and comorbidities, no strict frailty definition but more a physician-perceived frailty status based on clinical status and comorbidities [20, 24, 38].

The pooled result was an HR of 2.16 (95% CI: 1.56–3.00). Heterogeneity analysis showed that there was a significant difference in between-study variance (*I*^2^: 86%, τ^2^ = 0.1915, *p* < 0.01) (Fig. 2e).

### Anaemia

Five studies [53–57] (3746 patients) were included in the analysis for anaemia using the predefined cut-off values. The pooled result was an HR of 2.09 (95% CI: 0.93–4.66) and was not a significant predictor for 1‑year mortality, but nevertheless showed a clear trend. Heterogeneity analysis showed that there was a significant difference in between-study variance (*I*^2^: 95%, τ^2^ = 0.7797, *p* < 0.01) (Fig. 2f).

### Gait speed and ADL independence

For both gait speed [49, 58, 59] and ADL independence [49, 60] only a limited number of articles could be included in the multivariate 1‑year mortality analysis. For gait speed three studies were included in the meta-analysis (1501 patients) with a pooled HR of 13.35 (95% CI: 1.75–101.69). Heterogeneity analysis showed that there was a significant difference in between-study variance (*I*^2^: 94%, τ^2^ = 2.9070, *p* < 0.01) (Fig. 2g). For patients known to have any deficiency on the Katz ADL scale compared to those who did not, we included two studies, resulting in a pooled HR of 5.17 (95% CI: 0.77–34.57) and heterogeneity analysis showed that there was a significant difference in between-study variance (*I*^2^: 93%, τ^2^ = 1.7591, *p* < 0.01) (Fig. 2h).

**Fig. 3:**
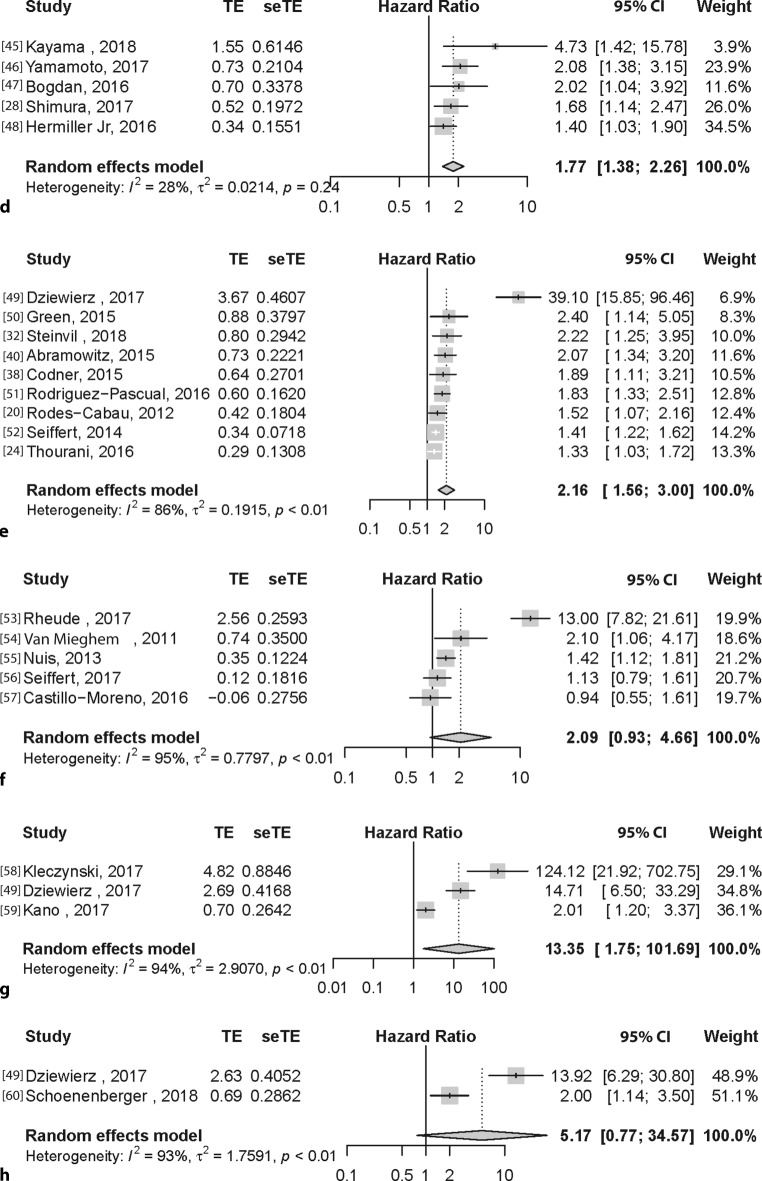
Forest plot summarising all variables. *eGFR* estimated glomerular filtration rate, *BMI* body mass index, *ADL* activities of daily living

Fig. 3 shows the summarised HRs of the different frailty subthemes, but these were not pooled together as the categories are not comparable. The strongest predictor of 1‑year mortality was low gait speed, but this also had the largest confidence interval: HR 13.33, 95% CI: 1.75–101.49. Overall the HRs ranged from 1.49 to 13.33.

## Discussion

In this systematic review and meta-analysis we studied the relationship between multiple components considered as preoperative frailty parameters, aggregated frailty scores and all-cause 1‑year mortality. Despite the fact that it is difficult to compare different subthemes of frailty we found that the most studied comorbidity is CLD, followed by CKD and underweight (BMI <20 kg/m^2^). Multiple comorbidities showed a strong association with worse 1‑year mortality. Surgical risk scores are mostly used to differentiate between patients eligible for surgical aortic valve repair. In the first step of this systematic review, where we identified frailty characteristics used in RCTs, we focused on the manifestation of frailty, which is often described as diminished physical functioning (due to skeletal muscle loss of sarcopenia and osteopenia) and a decrease in cognitive functioning [61]. In our review we used frailty parameters which are measurable and can be easily used in daily clinical practice. The novelty of this study is that it is a comprehensive review and consisted of a two-step approach in which variables used as a surrogate for frailty in RCTs in TAVI patients were employed.

Other reviews have focused on the added value of assessing frailty in TAVI patients and the association with outcome after TAVI [62] or focused on pooling studies with a frailty assessment [63]. In our two-step approach we first identified subthemes within the frailty domain from RCTs in TAVI patients to include in our search comorbidities which are often seen as a parameter of the aggregated frailty term.

### Frailty scores

Multiple frailty scores were used in the articles included in our pooled analysis. An earlier meta-analysis showed a clear association between dichotomised frailty scores and mortality after TAVI [63], but due to the differences in pooled studies and the structure of scales, meta-analysis and pooling of the results of non-dichotomised scales was not possible. Anand et al. also differentiated between subjective and objective assessment and found that both had a clear association with late (>30 days after TAVI) mortality [63]. Since some of the frailty scores used were a physician-based interpretation or an ‘eyeballing’ test, the implementation and objective scoring is challenging.

The frailty syndrome has an overlap with (co)morbidities and disability [64] and the parameters used often reflect a combination of those.

### CLD and CKD

Most articles reported the presence of any type and severity of CLD and not necessarily based on pulmonary function testing or the effect of the lung disease on the actual physical functioning. CKD was defined by an eGFR <30 ml/min. Previous research has shown that although an eGFR <60 ml/min is already considered to be CKD, the group below <30 ml/min had a significant impact on 1‑year mortality after TAVI [65].

### Malnutrition (underweight and/or hypoalbuminaemia)

In our analysis we used underweight and hypoalbuminaemia as indicators for malnutrition. Overweight and obesity can also be considered as malnutrition categories [44]. There was, however, only limited data about obesity and, consistent with the literature on cardiothoracic surgery, the obesity paradox might also be valid for TAVI patients, as slight overweight is protective for mortality, whereas obesity is not. A meta-analysis on the effect of BMI on outcomes after TAVI found that 30-day mortality was not related to underweight, whereas long-term all-cause mortality was. Results in overweight patients (BMI >25) were similar to those in patients with normal weight. Obese patients (BMI >30) showed a significantly better long-term survival [66].

### Anaemia

Anaemia was associated with worse outcomes after TAVI. A study not included in the meta-analysis, as the authors did not report on adjusted HR for 1‑year mortality, but that is worth mentioning, is that by DeLarochellière et al., who reported that in their TAVI cohort two out of three patients had a preprocedural anaemia, of which 90.4% had a potentially correctable cause. However, only a limited number of patients received therapy [67]. In our results we also show that anaemia is associated with worse 1‑year outcomes with a 77% increased risk.

### ADL deficiencies and low gait speed

For the two categories ADL deficiencies and gait speed we could include only a limited number of studies. Furthermore, there was a significant heterogeneity, making the results disputable. The Katz ADL score is a simple scoring tool which counts the number of deficits for six self-caring categories (e.g. dressing, feeding, mobility). Patients were completely independent if they needed assistance in none of the categories. Another approach would be to take disability (as the inverse of independence) [62]. One type of disability might influence outcomes more than another and more in-depth research in disability is warranted.

### Other literature

Anand et al. showed in their meta-analysis that objective frailty assessment identifies a group of more vulnerable patients than the ‘end-of-the-bed’ (or ‘eyeballing’) subjective assessment [63]. In our meta-analysis we did not include in the aggregated frailty category all of the articles we found by Anand et al., since the 1‑year mortality was not always reported or the definition used was already covered by another of our frailty subthemes.

### Limitations

Our study has several limitations. First, no studies randomised between frail and non-frail patients; patient selection in the reported studies might already be biased by underlying and unmeasured frailty, as TAVI patients are already considered a frail population. Second, we focused only on 1‑year mortality; however frailty and comorbidities are likely also to influence longer-term mortality and morbidity. Thirdly, in our study we used dichotomous outcome variables from TAVI RCTs, instead of using the continuous scales. However, this facilitated the harmonisation of and increased the comparability of studies. We therefore included articles with the most common cut-off values, leading to the exclusion of articles with different cut-offs and those reporting continuous variables, as there is great variation between studies as regards study design and end-point definitions. Variables possibly serving as frailty characteristics but not used as frailty derivatives in TAVI RCTs, such as non-transfemoral access, were not studied in this review, which is a limitation.

In several categories (underweight, low frailty score, anaemia, low gait speed and ADL deficiency) a heterogeneity was observed and reflects the variance in results, thus supporting the need for larger prospective studies in the future.

### Future perspectives

In order to better predict outcomes after TAVI and enhance patient selection, a dedicated TAVI risk score should be developed that incorporates the individual frailty parameters we pooled in this meta-analysis. Most of these parameters (kidney function, lung function, underweight, anaemia, hypoalbuminaemia) are already collected in standard routine TAVI care and therefore do not cause an extra burden to assess. The physical components gait speed and ADL independence might require more effort, but we show that they are predictive for outcome.

Recognising frail patients may facilitate identification of vulnerable patients and can lead to more patient-tailored disease management. The score used should be procedure-specific, as individual components of frailty might have a different weighting in total frailty dependent on the type of procedure a patient undergoes. Therefore, it is important to analyse the individual components or frailty parameters specifically for TAVI to come up with a TAVI-specific risk score. Thus frailty as a composite score should not be advocated to be part of a TAVI risk score, but the individual parameters of frailty contributing to outcomes should be taken because the relation between frailty and outcome is not a universal standard. These parameters can be differentiated between fixed and non-fixed risk factors based on the reversibility of the parameter. Prospective studies may be set up to investigate if an intervention to treat, for example, hypoalbuminaemia or physical training before the procedure improves outcomes.

## Conclusion

In this meta-analysis we identified multiple frailty parameters used in TAVI research which were predictive for 1‑year mortality. Chronic lung disease, chronic kidney disease, underweight, hypoalbuminaemia, a low frailty score, anaemia, low gait speed and an ADL deficiency were all associated with worse 1‑year outcomes. Further research into the combination of these factors may help to more completely identify specific patients at risk when undergoing TAVI procedures.

## Caption Electronic Supplementary Material

The Electronic Supplementary Materials covers: 1. the description of the found frailty characteristics and their synonyms, 2. full search strategy and 3) the baseline characteristics and quality of the included studies.
