# Supplementary material for: Preoperative frailty parameters as predictors for outcomes after transcatheter aortic valve implantation: a systematic review and meta-analysis
Source: Neth Heart J. 2020 Mar 18;28(5):280–92. doi: 10.1007/s12471-020-01379-0 (PMC7190780; doi:10.1007/s12471-020-01379-0)
Supplement: Supplementary file 1 — The Electronic Supplementary Materials covers: 1. the description of the found frailty characteristics and their synonyms, 2. full search strategy and 3) the baseline characteristics and quality of the included studies. [file 12471_2020_1379_MOESM1_ESM.docx]

# Supplementary materials

| **Characteristics** | **Synonyms** |  |  |  |
| --- | --- | --- | --- | --- |
| **Frailty** | vulnerability | |  |  |
| **Osteoporosis [MeSH]** | Osteoporoses | | Bone loss |  |
| **Gait speed** | Walking speed [MeSH] | | Walking pace | walk time |
| **5-m gait speed** | Walking speed [MeSH] | | Gait speed | walk time |
| **6 MWT** | six minute walk test | | SMWT | six minute walking test |
| **Home oxygen** | - | |  |  |
| **Charlson comordity** | Comorbidity | |  |  |
| **Grip strength** | hand strength [MeSH] | | grasps |  |
| **Lung diffusion capacity** | diffusion capacity | |  |  |
| **Malnutrition [MeSH]** | Nutritional deficiency | | Undernutrition | Malnourishment |
| **BMI** | Body Mass Index (MeSH) | | Quetelet Index |  |
| **Unplanned Weight loss** | Weight reduction | |  |  |
| **Anemia [MeSH]** | - | |  |  |
| **Anemia with previous transfusion** | anemia requiring transfusion | |  |  |
| **Albumin** | Serum albumin | | Plasma albumin | Hypoalbuminemia |
| **Falls** | Accidental falls (MeSH] | | Recent falls | Falls in past 6 months |
| **Wheelchair bound** | - | |  |  |
| **Bedbound** | - | |  |  |
| **Mobility** | Mobility limitation | | Ambulation difficulty | Ambulatory difficulties |
| **KATZ** | - | |  |  |
| **ADL** | Activities of Daily Living | | Self care |  |
| **MMSE** | Mini mental state examination | |  |  |
| **Geriatric status scale** | - | |  |  |
| **Independent living** | - | |  |  |
| **Dementia [MeSH]** | - | |  |  |
| **SPPB** | Short Physical Performance battery | |  |  |
| **Chair stand test** |  | |  |  |
| **Timed up and go test** |  | |  |  |

S1: Frailty characteristics found in TAVI RCTs

S2: Search

Database(s): **Ovid MEDLINE(R) Epub Ahead of Print, In-Process & Other Non-Indexed Citations, Ovid MEDLINE(R) Daily and Ovid MEDLINE(R)**1946 to Present 
Search Strategy: **2018-04-12**

| **#** | **Searches** | **Results** |
| --- | --- | --- |
| 1 | transcatheter aortic valve replacement/ | 2337 |
| 2 | (TAVI or T-AVI or TA-ViVI or TAViVI or TA-AVI or TF-AVI or TFAVI or TAP-API or TAVR or TAVRs or TA-AVR or TFAVR or TF-AVR or TV-AVI or TVAVI or TAOAVI* or TAO-AVI* or TV-AVR* or TVAVR* or PAVI or P-AVI or PAVR or P-AVR).tw,ot,kf. | 4932 |
| 3 | (TATM or T-AVR or THV).tw,kf. and (aort* or (heart valve adj2 (implant* or replac*))).mp. | 216 |
| 4 | ((percutan* or per-cutan* or transcutan* or trans-cutan* or transcath* or trans-cath* or transapic* or trans-apic* or transfemor* or trans-femor* or transsubclav* or subclav* or transaort* or trans-aort* or transvasc* or transvasc*) adj4 (AVR or AVI or AVRs or AVIs)).tw,kf. | 133 |
| **5** | **or/1-4 [TAVI I]** | **5787** |
| 6 | heart valve prosthesis implantation/ and (percutan* or per-cutan* or transcutan* or trans-cutan* or transcath* or trans-cath* or transapic* or trans-apic* or transfemor* or trans-femor* or transsubclav* or subclav* or transaort* or trans-aort* or transvasc* or transvasc*).mp. | 5440 |
| 7 | ((percutan* or per-cutan* or transcutan* or trans-cutan* or transcathet* or trans-cathet* or transapic* or apic* or transfemor* or trans-femor* or transsubclav* or trans-subclav* or transaort* or trans-aort* or transvasc* or trans-vasc* or transarter* or trans-arter*) adj9 (valve or valvular or valves or prosth* or bioprost*) adj3 (implant* or replac* or intervent* or insert* or repair* or surger*)).tw,kf. | 9713 |
| 8 | (CoreValve* or Core-Valve* or Jenavalve* or ASCTM or Edward* Sapien* or (Sapien* adj2 valv*) or Portico or Lotus).tw,ot,kf,hw. | 4565 |
| **9** | **or/6-8 [transcathether route or valves]** | **13586** |
| 10 | aortic valve stenosis/ | 21726 |
| 11 | aortic valve/ | 27502 |
| 12 | ((aort* or transaort*) adj6 (steno* or valv* or bioprosthes* or prosthes* or regurg*)).tw,kf. | 58999 |
| **13** | **or/10-12 [aorta valve/stenosis]** | **69055** |
| **14** | **9 and 13 [TAVI II]** | **8432** |
| **15** | **5 or 14 [TAVI I II]** | **9037** |
| 16 | animals/ not humans/ | 4413281 |
| **17** | **15 not 16 [HUMAN TAVI]** | **8918** |
| 18 | frail elderly/ or geriatric assessment/ or geriatrics/ or geriatricians/ | 58054 |
| 19 | (frail* or CFS* or CSHA or ISAR or feeble or vulnerabilit* or fragility or ((vulnerab* or fragil* or weak or weaker) adj6 (patient* or elderly or old* or comorbi* or age or aged or population* or group* or subgroup* or individual* or person* or physiolog*)) or geriat* or CGA or MGA or ((multidimensional* or multi-dimensional*) adj3 (asses* or prognost*))).tw,kf. | 181466 |
| 20 | mental status schedule/ or cognition/ or comprehension/ or neurocognitive disorders/ or cognition disorders/ or cognitive dysfunction/ or dementia/ or alzheimer disease/ or exp dementia, vascular/ | 266880 |
| 21 | (mini mental state or MMSE or cognit* or dement* or alzheimer).tw,kf. | 403346 |
| 22 | exp osteoporosis/ or exp bone resorption/ or bone density/ or absorptiometry, photon/ | 117563 |
| 23 | (osteopor* or osteopen* or (bone adj3 (densit* or loss* or resorpt*)) or BMD or DEXA or DXA or DXAscan or dual-energy X-ray* or absorptiometr*).tw,kf. | 143187 |
| 24 | accidental falls/ or disability evaluation/ or neuropsychological tests/ or disabled persons/ or exp gait disorders, neurologic/ or mobility limitation/ or self-help devices/ or wheelchairs/ or exp muscle strength/ or muscle, skeletal/ or psoas muscles/ or muscle weakness/ or exp muscular atrophy/ or exp walking/ or walk test/ or physical fitness/ or psychomotor performance/ or motor activity/ or accelerometry/ or mental competency/ or personal autonomy/ or self care/ or exp "activities of daily living"/ or Karnofsky performance status/ or assisted living facilities/ or homes for the aged/ | 633507 |
| 25 | (MFS or ((recent or past or previous or months or accidental or scale* or scor* or slip*) adj6 fall*)).tw,kf. | 12196 |
| 26 | (grip or gripping or handgrip* or (hand adj3 (strength* or grasp*)) or (grasp* adj3 (force* or strength* or power)) or psoa* or sarcopen* or musc* strength* or (skeletal muscle* adj3 (mass* or index or indices or measurement*)) or TPA or SMI or SMIs or walk* or 6MWD or 6MWT* or MWD or MWT* or SMWT or 5MWT* or 5MWD* or gait*).tw,kf. | 199432 |
| 27 | (((mobility or immobility) not ((leaflet* or cusp*) adj5 (mobility or immobil*))) or EMS or ((poor or slow or diminish* or impaired or limit* or low* or dimension* or physical or accelomet* or elderly or aged or scale or scales or score or scores or index or indices or status or instrumental or extended or Duke*1) adj3 (mobil* or ambulat* or activit*))).tw,kf. | 370276 |
| 28 | (autonomy or (self adj (sufficien* or car* or relian* or directi*)) or wheelchair* or wheel-chair* or chair-bound or chairbound or chairfast or rollator* or ambulation aid* or (stick* adj3 (depend* or assist*)) or cane or canes or crutch* or bedbound or bed-bound or bed-ridden or bedridden or bed-confined or bedfast or independenc* or ((dependen* or independen*) adj1 functional*) or ((functional* or index or indices or scor*) adj2 (independenc* or dependenc*)) or ((living or life or activit* or home or soci*) adj2 (assisted or independen* or dependen* or participat*)) or community dwell* or disabilit* or disabled or disablement* or handicap* or SPPB or (performance adj1 (status or battery or based function* or scale* or physical)) or Fried criter* or ((daily or daytime or usual or count*) adj2 (activit* or life or living)) or slowness or ADL or ADLs or IADL or Katz or Barthel or Frenchay* or Lawton* or Nottingham or Karnofsky or "timed up and go" or chair stand*).tw,kf. | 438982 |
| 29 | nutrition assessment/ or malnutrition/ or overnutrition/ or nutritional physiological phenomena/ or elder nutritional physiological phenomena/ or nutritional status/ or exp energy intake/ or fasting/ or portion size/ or serving size/ or body mass index/ or body surface area/ or skinfold thickness/ or waist-hip ratio/ or adipose tissue/ or exp abdominal fat/ or body composition/ or exp body fat distribution/ or overweight/ or obesity/ or obesity, abdominal/ or obesity, morbid/ or exp body weight changes/ or thinness/ | 481223 |
| 30 | (nutrition* or calori* or malnutrit* or undernutrit* or underfeed* or malnourish* or undernourish* or overnutrit* or overnourish* or food-intake or quetelet or skinfold or waist-hip or waist-circumfer* or body weight or fat mass* or underweight* or overweight* or cachex* or cachect* or thinnes* or leanness* or obes* or adipos*).tw,kf. or body surface area*.ti. or (((weight or BW or BMI or body mass*) adj3 (low* or loss*)) not ((birth* or neonat* or infant* or molecular) adj3 weight*)).tw,kf. or (body mass* or fat mass* or body surfac* or BMI or body weight*).ti,kf. | 871499 |
| 31 | anemia/ or *blood transfusion/ or hemoglobins/df or hematocrit/ | 102145 |
| 32 | ((an?em* not (h?emolytic adj3 an?em*)) or ((low* or deficien*) adj3 (h?emoglobin* or h?ematocrit or iron)) or ((previous or earlier or histor* or baseline or pre-operat* or preoperat* or presurg* or pre-surg* or preTAVI or pre-TAVI or preprocedur* or pre-procedur* or associat* or correlat*) adj8 blood adj1 transfus*)).tw,kf. | 153708 |
| 33 | serum albumin/ or hypoalbuminemia/ | 46257 |
| 34 | (((serum or low* or level* or measur* or baseline or pre-operat* or preoperat* or presurg* or pre-surg* or preTAVI or pre-TAVI or preprocedur* or pre-procedur*) adj3 albumin*) or hypoalbumin* or hypo-albumin*).tw,kf. | 79847 |
| 35 | lung diseases/ or lung diseases, obstructive/ or asthma/ or exp pulmonary disease, chronic obstructive/ or exp pulmonary gas exchange/ or respiratory function tests/ or exp lung volume measurements/ or spirometry/ | 306305 |
| 36 | (((chronic or obstruct* or restrictive or severe or advanced or oxygen-dependent or pre-exist* or baseline or pre-operat* or preoperat* or presurg* or pre-surg* or preTAVI or pre-TAVI or preprocedur* or pre-procedur*) adj3 (lung or pulmonary or airway or airflow) adj diseas*) or chronic bronchit* or COPD or COAD or asthma* or (home adj3 oxygen) or ((lung or pulmon* or capacit*) adj3 diffus*) or ((respirat* or lung or pulmonar*) adj3 function test*) or spirometr* or vital capacit* or total lung capacit* or forced expiratory volum* or FVC or FEV or FEV1).tw,kf. | 254983 |
| 37 | *comorbidity/ or ((comorbidity/ or health status/) and (*patient selection/ or medical futility/ or (((risk or age or patient*) adj3 stratif*) or futil*).tw,kf. or patient select*.ti,kf.)) | 4563 |
| 38 | (comorbid* or co-morbid* or multimorb* or multi-morb*).ti,ot. or (Charlson* or Elixhauser* or ((comorbidit* or co-morbidit* or multimorb* or multi-morb*) adj2 (index or indices or scor* or degree* or number* or profile* or asses* or measure* or determin* or rate or rating or test* or exam*)) or high comorbidit* or cumulativ* ill* or CIRS or ((coexistent or chronic) adj2 (condition* or illness*))).tw,kf. | 73408 |
| **39** | **or/18-38 [FRAILTY-parameters]** | **3565326** |
| **40** | **17 and 39 [ II TAVI-FRAILTY]** | **808** |
| **41** | **remove duplicates from 40 [ II TAVI-FRAILTY -deduplicated]** | **804** |

Database(s): **Embase Classic+Embase**1947 to 2018 April 11 
Search Strategy: **2018-04-12**

| **#** | **Searches** | **Results** |
| --- | --- | --- |
| 1 | transcatheter aortic valve implantation/ | 14836 |
| 2 | (TAVI or T-AVI or TA-ViVI or TAViVI or TA-AVI or TF-AVI or TFAVI or TAP-API or TAVR or TAVRs or TA-AVR or TFAVR or TF-AVR or TV-AVI or TVAVI or TAOAVI* or TAO-AVI* or TV-AVR* or TVAVR* or PAVI or P-AVI or PAVR or P-AVR).tw,ot,kw. | 11881 |
| 3 | (TATM or T-AVR or THV).tw,kw. and (aort* or (heart valve adj2 (implant* or replac*))).mp. | 457 |
| 4 | ((percutan* or per-cutan* or transcutan* or trans-cutan* or transcath* or trans-cath* or transapic* or trans-apic* or transfemor* or trans-femor* or transsubclav* or subclav* or transaort* or trans-aort* or transvasc* or transvasc*) adj4 (AVR or AVI or AVRs or AVIs)).tw,kw. | 333 |
| **5** | **or/1-4 [TAVI I]** | **16580** |
| 6 | percutaneous aortic valve/ | 2229 |
| 7 | ((percutan* or per-cutan* or transcutan* or trans-cutan* or transcathet* or trans-cathet* or transapic* or apic* or transfemor* or trans-femor* or transsubclav* or trans-subclav* or transaort* or trans-aort* or transvasc* or trans-vasc* or transarter* or trans-arter*) adj9 (valve or valvular or valves or prosth* or bioprost*) adj3 (implant* or replac* or intervent* or insert* or repair* or surger*)).tw,kw. | 18075 |
| 8 | (CoreValve* or Core-Valve* or Jenavalve* or ASCTM or Edward* Sapien* or (Sapien* adj2 valv*) or Portico or Lotus).tw,ot,kw,hw. | 7910 |
| 9 | or/7-8 [transcathether route or valves] | 21690 |
| 10 | aorta valve stenosis/ | 12972 |
| 11 | aorta valve/ or aorta valve replacement/ | 39529 |
| 12 | ((aort* or transaort*) adj6 (steno* or valv* or bioprosthes* or prosthes* or regurg*)).tw,kw. | 89288 |
| 13 | or/10-12 [aorta valve/stenosis] | 99591 |
| **14** | **9 and 13 [TAVI II]** | **15329** |
| **15** | **5 or 14 [TAVI I II]** | **18445** |
| 16 | (animal.hw. or nonhuman/) not human/ | 5903043 |
| **17** | **15 not 16 [HUMAN TAVI]** | **18233** |
| 18 | frail elderly/ or geriatric disorder/ or frailty/ or geriatric assessment/ or geriatrician/ | 27984 |
| 19 | (frail* or CFS* or CSHA or ISAR or feeble or vulnerabilit* or fragility or ((vulnerab* or fragil* or weak or weaker) adj6 (patient* or elderly or old* or comorbi* or age or aged or population* or group* or subgroup* or individual* or person* or physiolog*)) or geriat* or CGA or MGA or ((multidimensional* or multi-dimensional*) adj3 (asses* or prognost*))).tw,kw. | 247784 |
| 20 | cognition/ or cognition assessment/ or cognitive defect/ or comprehension/ or dementia/ or alzheimer disease/ or multiinfarct dementia/ or presenile dementia/ or exp senile dementia/ or dementia assessment/ or alzheimer disease assessment scale/ or clinical dementia rating/ or mini mental state examination/ or geriatric depression scale/ or cornell scale for depression in dementia/ | 557986 |
| 21 | (mini mental state or MMSE or cognit* or dement* or alzheimer).tw,kw. | 569763 |
| 22 | exp bone demineralization/ or osteolysis/ or exp bone density/ or dual energy x ray absorptiometry/ | 223511 |
| 23 | (osteopor* or osteopen* or (bone adj3 (densit* or loss* or resorpt*)) or BMD or DEXA or DXA or DXAscan or dual-energy X-ray* or absorptiometr*).tw,kw. | 207360 |
| 24 | patient mobility/ or physical mobility/ or disability/ or exp adl disability/ or physical disability/ or invalidity/ or disabled person/ or limited mobility/ or walking difficulty/ or gait disorder/ or exp walking aid/ or exp wheelchair/ or exp hand grip/ or muscle mass/ or muscle strength/ or skeletal muscle/ or psoas muscle/ or muscle atrophy/ or sarcopenia/ or exp walking/ or exp walk test/ or motor dysfunction assessment/ or dynamic gait index/ or falls efficacy scale/ or morse fall scale/ or performance oriented mobility assessment/ or rivermead mobility index/ or "timed up and go test"/ or "physical activity, capacity and performance"/ or physical capacity/ or physical inactivity/ or exp physical performance/ or accelerometry/ or pedometry/ or exhaustion/ or weakness/ or daily life activity/ or independent living/ or "activity of daily living assessment"/ or karnofsky performance status/ or self care/ or assisted living facility/ or home for the aged/ | 741446 |
| 25 | (MFS or ((recent or past or previous or months or accidental or scale* or scor* or slip*) adj6 fall*)).tw,kw. | 18109 |
| 26 | (grip or gripping or handgrip* or (hand adj3 (strength* or grasp*)) or (grasp* adj3 (force* or strength* or power)) or psoa* or sarcopen* or musc* strength* or (skeletal muscle* adj3 (mass* or index or indices or measurement*)) or TPA or SMI or SMIs or walk* or 6MWD or 6MWT* or MWD or MWT* or SMWT or 5MWT* or 5MWD* or gait*).tw,kw. | 283127 |
| 27 | (((mobility or immobility) not ((leaflet* or cusp*) adj5 (mobility or immobil*))) or EMS or ((poor or slow or diminish* or impaired or limit* or low* or dimension* or physical or accelomet* or elderly or aged or scale or scales or score or scores or index or indices or status or instrumental or extended or Duke*1) adj3 (mobil* or ambulat* or activit*))).tw,kw. | 494137 |
| 28 | (autonomy or (self adj (sufficien* or car* or relian* or directi*)) or wheelchair* or wheel-chair* or chair-bound or chairbound or chairfast or rollator* or ambulation aid* or (stick* adj3 (depend* or assist*)) or cane or canes or crutch* or bedbound or bed-bound or bed-ridden or bedridden or bed-confined or bedfast or independenc* or ((dependen* or independen*) adj1 functional*) or ((functional* or index or indices or scor*) adj2 (independenc* or dependenc*)) or ((living or life or activit* or home or soci*) adj2 (assisted or independen* or dependen* or participat*)) or community dwell* or disabilit* or disabled or disablement* or handicap* or SPPB or (performance adj1 (status or battery or based function* or scale* or physical)) or Fried criter* or ((daily or daytime or usual or count*) adj2 (activit* or life or living)) or slowness or ADL or ADLs or IADL or Katz or Barthel or Frenchay* or Lawton* or Nottingham or Karnofsky or "timed up and go" or chair stand*).tw,kw. | 618507 |
| 29 | nutrition/ or geriatric nutrition/ or nutritional assessment/ or nutritional status/ or malnutrition/ or cachexia/ or *obesity/ or overnutrition/ or abdominal obesity/ or morbid obesity/ or sarcopenic obesity/ or lean body weight/ or weight change/ or exp weight gain/ or exp weight reduction/ or *body mass/ or *body surface/ or body composition/ or body distribution/ or exp abdominal fat/ or body fat/ or fat free mass/ or fat mass/ or skinfold thickness/ or waist hip ratio/ | 689710 |
| 30 | (nutrition* or calori* or malnutrit* or undernutrit* or underfeed* or malnourish* or undernourish* or overnutrit* or overnourish* or food-intake or quetelet or skinfold or waist-hip or waist-circumfer* or body weight or fat mass* or underweight* or overweight* or cachex* or cachect* or thinnes* or leanness* or obes* or adipos*).tw,kw. or body surface area*.ti. or (((weight or BW or BMI or body mass*) adj3 (low* or loss*)) not ((birth* or neonat* or infant* or molecular) adj3 weight*)).tw,kw. or (body mass* or fat mass* or body surfac* or BMI or body weight*).ti,kw. | 1230254 |
| 31 | *anemia/ or *blood transfusion/ or hematocrit/ | 137954 |
| 32 | ((an?em* not (h?emolytic adj3 an?em*)) or ((low* or deficien*) adj3 (h?emoglobin* or h?ematocrit or iron)) or ((previous or earlier or histor* or baseline or pre-operat* or preoperat* or presurg* or pre-surg* or preTAVI or pre-TAVI or preprocedur* or pre-procedur* or associat* or correlat*) adj8 blood adj1 transfus*)).tw,kw. | 234240 |
| 33 | hypoalbuminemia/ or serum albumin/ or albumin blood level/ | 66784 |
| 34 | (((serum or low* or level* or measur* or baseline or pre-operat* or preoperat* or presurg* or pre-surg* or preTAVI or pre-TAVI or preprocedur* or pre-procedur*) adj3 albumin*) or hypoalbumin* or hypo-albumin*).tw,kw. | 107332 |
| 35 | *lung disease/ or *chronic lung disease/ or *chronic obstructive lung disease/ or asthma/ or chronic bronchitis/ or home oxygen therapy/ or lung function test/ or lung diffusion capacity/ or spirometry/ or expiratory reserve volume/ or functional residual capacity/ or lung capacity/ or vital capacity/ | 390873 |
| 36 | (((chronic or obstruct* or restrictive or severe or advanced or oxygen-dependent or pre-exist* or baseline or pre-operat* or preoperat* or presurg* or pre-surg* or preTAVI or pre-TAVI or preprocedur* or pre-procedur*) adj3 (lung or pulmonary or airway or airflow) adj diseas*) or chronic bronchit* or COPD or COAD or asthma* or (home adj3 oxygen) or ((lung or pulmon* or capacit*) adj3 diffus*) or ((respirat* or lung or pulmonar*) adj3 function test*) or spirometr* or vital capacit* or total lung capacit* or forced expiratory volum* or FVC or FEV or FEV1).tw,kw. | 398054 |
| 37 | *comorbidity/ or exp comorbidity assessment/ or multiple chronic conditions/ | 24190 |
| 38 | (comorbid* or co-morbid* or multimorb* or multi-morb*).ti,ot. or (Charlson* or Elixhauser* or ((comorbidit* or co-morbidit* or multimorb* or multi-morb*) adj2 (index or indices or scor* or degree* or number* or profile* or asses* or measure* or determin* or rate or rating or test* or exam*)) or high comorbidit* or cumulativ* ill* or CIRS or ((coexistent or chronic) adj2 (condition* or illness*))).tw,kw. | 115772 |
| **39** | **or/18-38 [FRAILTY-parameters]** | **4785098** |
| **40** | **17 and 39 [TAVI-FRAILTY]** | **2188** |
| **41** | **remove duplicates from 40** | **2128** |
| 42 | (embase or elsevier or canadian).cr. | 25108623 |
| **43** | **41 and 42** | **2092** |
| 44 | editorial/ or (conference abstract or "conference review" or editorial or note).pt. | 4292530 |
| **45** | **43 not 44 [TAVI-FRAILTY deduplicated EMBASE records not conference/editorials]** | **907** |

RCT search

Database(s): **Ovid MEDLINE(R) Epub Ahead of Print, In-Process & Other Non-Indexed Citations, Ovid MEDLINE(R) Daily and Ovid MEDLINE(R)**1946 to Present 
Search Strategy: **2018-04-12**

| 1 | transcatheter aortic valve replacement/ | 2337 |
| --- | --- | --- |
| 2 | (TAVI or T-AVI or TA-ViVI or TAViVI or TA-AVI or TF-AVI or TFAVI or TAP-API or TAVR or TAVRs or TA-AVR or TFAVR or TF-AVR or TV-AVI or TVAVI or TAOAVI* or TAO-AVI* or TV-AVR* or TVAVR* or PAVI or P-AVI or PAVR or P-AVR).tw,ot,kf. | 4932 |
| 3 | (TATM or T-AVR or THV).tw,kf. and (aort* or (heart valve adj2 (implant* or replac*))).mp. | 216 |
| 4 | ((percutan* or per-cutan* or transcutan* or trans-cutan* or transcath* or trans-cath* or transapic* or trans-apic* or transfemor* or trans-femor* or transsubclav* or subclav* or transaort* or trans-aort* or transvasc* or transvasc*) adj4 (AVR or AVI or AVRs or AVIs)).tw,kf. | 133 |
| 5 | or/1-4 [TAVI I] | 5787 |
| 6 | heart valve prosthesis implantation/ and (percutan* or per-cutan* or transcutan* or trans-cutan* or transcath* or trans-cath* or transapic* or trans-apic* or transfemor* or trans-femor* or transsubclav* or subclav* or transaort* or trans-aort* or transvasc* or transvasc*).mp. | 5440 |
| 7 | ((percutan* or per-cutan* or transcutan* or trans-cutan* or transcathet* or trans-cathet* or transapic* or apic* or transfemor* or trans-femor* or transsubclav* or trans-subclav* or transaort* or trans-aort* or transvasc* or trans-vasc* or transarter* or trans-arter*) adj9 (valve or valvular or valves or prosth* or bioprost*) adj3 (implant* or replac* or intervent* or insert* or repair* or surger*)).tw,kf. | 9713 |
| 8 | (CoreValve* or Core-Valve* or Jenavalve* or ASCTM or Edward* Sapien* or (Sapien* adj2 valv*) or Portico or Lotus).tw,ot,kf,hw. | 4565 |
| 9 | or/6-8 [transcathether route or valves] | 13586 |
| 10 | aortic valve stenosis/ | 21726 |
| 11 | aortic valve/ | 27502 |
| 12 | ((aort* or transaort*) adj6 (steno* or valv* or bioprosthes* or prosthes* or regurg*)).tw,kf. | 58999 |
| 13 | or/10-12 [aorta valve/stenosis] | 69055 |
| 14 | 9 and 13 [TAVI II] | 8432 |
| 15 | 5 or 14 [TAVI I II] | 9037 |
| 16 | animals/ not humans/ | 4413281 |
| 17 | 15 not 16 [HUMAN TAVI] | 8918 |
| 18 | (controlled clinical trial/ or randomized controlled trial/ or random allocation/ or double-blind method/ or single-blind method/ or (randomi?ed or placebo* or randomly or (random adj3 allocat*) or ((random* or controlled) adj2 (study or trial)) or ((singl* or doubl* or treb* or tripl*) adj (blind*3 or mask*3))).tw,kf. or trial.ti.) not ((cochrane or systematic review* or clinical evidence or EBM).jw. or editorial/ or (systematic* adj3 (review or literature)).ti. or ((search* adj12 (literature* or ((electronic or medical or biomedical) adj3 database*) or exhaustiv* or systematic*)) or medline or pubmed or embase or psychinfo or (CENTRAL and cochrane) or "Central Register of Controlled Trials").tw. or ((review/ or meta-analysis/ or (meta analy* or metaanaly* or meta?analy*).ti,ot. or (systematic* adj3 (review or literature)).tw,kf.) not (controlled clinical trial/ or randomized controlled trial/ or ((random* and trial) or (controlled adj2 trial)).ti,ot.))) [Filter for RCTs not reviews or editorials] | 1007802 |
| 19 | 17 and 18 [ I human RCTs on TAVI] | 421 |
| **20** | **remove duplicates from 19 [human RCTs on TAVI -deduplicated ]** | **416** |

Database(s): **Epub Ahead of Print, In-Process & Other Non-Indexed Citations, Ovid MEDLINE(R) Daily and Ovid MEDLINE(R)**1946 to Present 
Search Strategy: **2016-09-23**

| **#** | **Searches** | **Results** |
| --- | --- | --- |
| 1 | Transcatheter Aortic Valve Replacement/ | 1070 |
| 2 | (TAVI or T-AVI or TA-ViVI or TAViVI or TA-AVI or TF-AVI or TFAVI or TAP-API or TAVR or TAVRs or TA-AVR or TFAVR or TF-AVR or TV-AVI or TVAVI or TAOAVI* or TAO-AVI* or TV-AVR* or TVAVR*).tw,ot,kf. | 3489 |
| 3 | (TATM or T-AVR or THV).tw,kf. and (aort* or (heart valve adj2 implant*)).mp. | 150 |
| 4 | ((percutan* or per-cutan* or transcutan* or trans-cutan* or transcath* or trans-cath* or transapic* or trans-apic* or transfemor* or trans-femor* or transsubclav* or subclav* or transaort* or trans-aort* or transvasc* or transvasc*) adj4 (AVR or AVI or AVRs or AVIs)).tw,kf. | 111 |
| **5** | **or/1-4 [TAVI I]** | **3978** |
| 6 | ((percutan* or per-cutan* or transcutan* or trans-cutan* or transcathet* or trans-cathet* or transapic* or apic* or transfemor* or trans-femor* or transsubclav* or trans-subclav* or transaort* or trans-aort* or transvasc* or trans-vasc* or transarter* or trans-arter*) adj9 (valve or valvular or valves or prosth* or bioprost*) adj3 (implant* or replac* or intervent* or insert* or repair* or surger*)).tw,kf. | 7297 |
| 7 | (CoreValve* or Core-Valve* or Jenavalve* or ASCTM or Edward* Sapien* or (Sapien* adj2 valv*) or Portico or Lotus).tw,ot,kf,hw. | 4005 |
| 8 | or/6-7 [transcathether route or valves] | 9957 |
| 9 | aortic valve stenosis/ | 20103 |
| 10 | aortic valve/ | 26590 |
| 11 | ((aort* or transaort*) adj6 (steno* or valv* or bioprosthes* or prosthes* or regurg*)).tw,kf. | 55092 |
| **12** | **or/9-11 [aorta valve/stenosis]** | **65077** |
| **13** | **8 and 12 [TAVI II]** | **5981** |
| **14** | **5 or 13 [TAVI I II]** | **6431** |
| **15** | **animals/ not humans/** | **4287472** |
| **16** | **14 not 15 [HUMAN TAVI]** | **6336** |
| 17 | (controlled clinical trial/ or randomized controlled trial/ or random allocation/ or double-blind method/ or single-blind method/ or (randomi?ed or placebo* or randomly or (random adj3 allocat*) or ((random* or controlled) adj2 (study or trial)) or ((singl* or doubl* or treb* or tripl*) adj (blind*3 or mask*3))).tw,kf. or trial.ti.) not ((cochrane or systematic review* or clinical evidence or EBM).jw. or editorial/ or (systematic* adj3 (review or literature)).ti. or ((search* adj12 (literature* or ((electronic or medical or biomedical) adj3 database*) or exhaustiv* or systematic*)) or medline or pubmed or embase or psychinfo or (CENTRAL and cochrane) or "Central Register of Controlled Trials").tw. or ((review/ or meta-analysis/ or (meta analy* or metaanaly* or meta?analy*).ti,ot. or (systematic* adj3 (review or literature)).tw,kf.) not (controlled clinical trial/ or randomized controlled trial/ or ((random* and trial) or (controlled adj2 trial)).ti,ot.))) [Filter for RCTs not reviews or editorials] | 941666 |
| **18** | **16 and 17 [RCTs on TAVI]** | **335** |
| **19** | **remove duplicates from 18** | **316** |

| **S3: Study demographics** | |  |  |  |  |  |  |  |  |  |  |  |
| --- | --- | --- | --- | --- | --- | --- | --- | --- | --- | --- | --- | --- |
| **Variable** | **Author** | **Year** | **n** | **Mean age (yr)** | **Sex**  **(male, %)** | **EuroSCORE I** | **Euro**  **SCORE II** | **STS** | **NOS**  **selection** | **NOS**  **comparability** | **NOS**  **exposure** | **NOS**  **total** |
| Anemia | M. Seiffert, et al.^56^ | 2017 | 847 | 80.9 | 48.9 | 20.4 | 5.6 | 6.4 | 3 | 2 | 3 | 8 |
| Anemia | J. A. Castillo-Moreno, et al.^57^ | 2016 | 182 | 75 | 50.3 | NR | NR | NR | 4 | 1 | 2 | 7 |
| Anemia | R. J. Nuis, et al.^55^ | 2013 | 1696 | 81 | 52 | 19 | NR | NR | 3 | 2 | 1 | 6 |
| Anemia | N. M. Van Mieghem, et al.^54^ | 2011 | 118 | 82 | 51 | 12.3 | NR | 6 | 4 | 1 | 1 | 6 |
| Anemia | T. Rheude, et al. ^53^ | 2017 | 549 | 81 | 55 | 13 | 4 | NR | 4 | 2 | 3 | 9 |
| eGFR <30 | N. E. Moat, et al.^31^ | 2011 | 870 | 81.9 | 52.4 | 18.5 | NR | NR | 3 | 2 | 2 | 7 |
| eGFR <30 | P. F. Ludman, et al.^4^ | 2015 | 3980 | 81.3 | 47.3 | 21.9 | NR | NR | 3 | 2 | 2 | 7 |
| eGFR <30 | P. Debonnaire, et al.^29^ | 2015 | 511 | 82 | 38.0 | 18.3 | 6,4 | 16,6 | 3 | 1 | 2 | 6 |
| eGFR <30 | F. Saia, et al.^41^ | 2014 | 874 | 80,7 | 47 | 23,4 | 7,1 | 9 | 3 | 2 | 2 | 7 |
| eGFR <30 | V. H. Thourani, et al.^24^ | 2016 | 2100 | 84,4 | 52,4 | NR | NR | 11,5 | 3 | 2 | 3 | 8 |
| eGFR <30 | K. Hemmann, et al.^36^ | 2013 | 426 | 80 | 48,0 | 19,5 | 7,8 | 7,4 | 3 | 1 | 3 | 7 |
| eGFR <30 | Y. Abramowitz. et al.^40^ | 2015 | 734 | 82.1 | 60.5 | NR | 10.3 | 8.1 | 3 | 2 | 2 | 7 |
| eGFR <30 | A. Steinvil. et al.^32^ | 2018 | 498 | 82 | 49 | NR | NR | 7.5 | 3 | 2 | 1 | 6 |
| Low frailty score | V. H. Thourani. et al.^24^ | 2016 | 2100 | 84.4 | 52.4 | NR | NR | 11.5 | 3 | 2 | 0 | 5 |
| Low frailty score | M. Seiffert. et al.^52^ | 2014 | 847 | 80.9 | 48.9 | 20.4 | 5.6 | 6.4 | 3 | 2 | 3 | 8 |
| Low frailty score | J. Rodes-Cabau. et al.^20^ | 2012 | 339 | 81 | 44.8 | NR | NR | 9.8 | 4 | 2 | 2 | 8 |
| Low frailty score | C. Rodriguez-Pascual. et al.^51^ | 2016 | 606 | 83.3 | 43.1 | 13.2 | NR | 9 | 2 | 2 | 1 | 5 |
| Low frailty score | P. Codner. et al.^38^ | 2015 | 360 | 82.1 | 43.6 | 19.5 | NR | 7.5 | 3 | 2 | 3 | 8 |
| Low frailty score | Y. Abramowitz. et al.^40^ | 2015 | 734 | 82.1 | 60.5 | NR | 10.3 | 8.1 | 2 | 1 | 2 | 5 |
| Low frailty score | P. Green. et al.^50^ | 2015 | 244 | NR | 51.6 | NR | NR | NR | 4 | 2 | 1 | 7 |
| Low frailty score | A. Dziewierz et al.^49^ | 2017 | 148 | 82 | 37.8 | 14.5 | NR | 6.2 | 3 | 2 | 1 | 6 |
| Low frailty score | A. Steinvil et al.^32^ | 2018 | 498 | 82 | 49 | NR | NR | 7.5 | 4 | 2 | 1 | 7 |
| CLD | E. Koifman. et al.^39^ | 2015 | 476 | 83.5 | 73.0 | NR | NR | 9.7 | 3 | 0 | 1 | 4 |
| CLD | S. Salizzoni. et al.^37^ | 2016 | 1904 | 81.7 | 39.8 | 21.1 | 7.3 | 9.2 | 4 | 2 | 3 | 9 |
| CLD | P. Codner. et al.^38^ | 2015 | 360 | 82.1 | 43.6 | 19.5 | NR | 7.5 | 3 | 2 | 1 | 6 |
| CLD | K. Hemmann. et al.^36^ | 2013 | 426 | 80 | 48.0 | 19.5 | 7.8 | 7.4 | 3 | 2 | 3 | 8 |
| CLD | E. A. Grossi. et al.^35^ | 2008 | 713 | NR | 49.9 | 17.2 | NR | NR | 3 | 0 | 2 | 5 |
| CLD | P. F. Ludman. et al.^4^ | 2015 | 3980 | 81.3 | 47.3 | 21.9 | NR | NR | 3 | 0 | 2 | 5 |
| CLD | D. R. Holmes. Jr.. et al.^33^ | 2015 | 12182 | 84 | 48.1 | NR | NR | 7.1 | 3 | 2 | 0 | 5 |
| CLD | N. E. Moat. et al.^31^ | 2011 | 870 | 81.9 | 52.4 | 18.5 | NR | NR | 3 | 2 | 1 | 6 |
| CLD | S. Bleiziffer. et al.^30^ | 2017 | 996 | 81.1 | 49.4 | 19.4 | NR | NR | 3 | 2 | 2 | 7 |
| CLD | P. Debonnaire. et al.^29^ | 2015 | 511 | 82 | 38.0 | 18.3 | 6.4 | 16.6 | 3 | 1 | 1 | 5 |
| CLD | T. Shimura. et al.^28^ | 2017 | 1215 | 84.4 | 29.7 | 17 | NR | NR | 3 | 2 | 2 | 7 |
| CLD | M. Urena. et al.^27^ | 2015 | 3726 | 81 | 50.2 | 19.4 | NR | NR | 3 | 1 | 3 | 7 |
| CLD | M. Konigstein. et al.^26^ | 2015 | 409 | 82 | 42.0 | 24 | NR | NR | 3 | 1 | 3 | 7 |
| CLD | V. H. Thourani. et al.^24^ | 2016 | 2100 | 84.4 | 52.4 | NR | NR | 11.5 | 3 | 2 | 3 | 8 |
| CLD | R. Gonzalez-Ferreiro. et al.^23^ | 2017 | 770 | 80.7 | 43.1 | 17.8 | NR | NR | 3 | 1 | 2 | 6 |
| CLD | J. Rodes-Cabau. et al.^68^ | 2010 | 339 | 81 | 44.8 | NR | NR | 9.8 | 3 | 2 | 2 | 7 |
| CLD | I. M. Barbash. et al.^21^ | 2015 | 415 | 84 | 47.0 | 30 | NR | 10 | 4 | 2 | 1 | 7 |
| CLD | M. Mok. et al.^19^ | 2013 | 319 | 80 | 46.1 | NR | NR | 6.3 | 3 | 2 | 2 | 7 |
| CLD | J. Rodes-Cabau. et al.^69^ | 2012 | 339 | 81 | 44.8 | NR | NR | 9.8 | 3 | 2 | 1 | 6 |
| CLD | A. J. Munoz-Garcia. et al.^18^ | 2013 | 1220 | 80.7 | 55.3 | 17.8 | NR | NR | 3 | 2 | 1 | 6 |
| CLD | M. Mok. et al.^15^ | 2016 | 460 | 81 | 51.0 | NR | NR | 6.9 | 3 | 1 | 2 | 6 |
| CLD | R. A. Meneguz-Moreno. et al.^14^ | 2017 | 221 | 82.24 | 46.6 | NR | 7.66 | 6.41 | 3 | 2 | 2 | 7 |
| CLD | F. S. de Brito. Jr.. et al.^13^ | 2015 | 418 | 81.5 | 47.8 | 20.2 | NR | 14.2 | 3 | 2 | 3 | 8 |
| CLD | MC Henn. et al.^16^ | 2016 | 246 | 81 | 51 | NR | NR | 10.4 | 3 | 2 | 3 | 8 |
| CLD | H. Hioki et al.^34^ | 2018 | 1613 | 85 | 29.6 | NR | NR | 6.66 | 3 | 2 | 0 | 5 |
| CLD | S.A. Hirji et al. ^17^ | 2017 | 306 | 86.2 | 47.8 | NR | NR | 6.81 | 2 | 2 | 3 | 7 |
| CLD | M. Lutz. et al.^22^ | 2017 | 217 | 82 | 44.2 | 20.1 | 6.1 | 5.2 | 2 | 2 | 2 | 6 |
| CLD | HB Ribeiro. et al.^25^ | 2018 | 287 | 80 | 72.1 | NR | 10.5 | 7.7 | 3 | 2 | 3 | 8 |
| CLD | A. Steinvil et al.^32^ | 2018 | 498 | 82 | 49 | NR | NR | 7.5 | 3 | 2 | 1 | 6 |
| Hypoalbuminemia | M. Yamamoto. et al.^46^ | 2017 | 1215 | 84.4 | 29.7 | 12.8 | NR | 6.8 | 4 | 2 | 2 | 8 |
| Hypoalbuminemia | J. B. Hermiller. Jr.. et al.^48^ | 2016 | 3687 | 83.3 | 53.7 | 22.2 | NR | NR | 3 | 0 | 2 | 5 |
| Hypoalbuminemia | T. Shimura. et al.^28^ | 2017 | 1215 | 84.4 | 29.7 | 17 | NR | NR | 3 | 1 | 1 | 5 |
| Hypoalbuminemia | A. Bogdan. et al.^47^ | 2016 | 150 | 81 | 40 | 17.27 | 5.72 | 5.49 | 4 | 2 | 2 | 8 |
| Hypoalbuminemia | S. Kayama. et al.^45^ | 2018 | 80 | 85 | 22.5 | 18.4 | 4.6 | 8.1 | 3 | 2 | 3 | 8 |
| BMI < 20 | T. Shimura. et al.^28^ | 2017 | 1215 | 84.4 | 29.7 | 17 | NR | NR | 3 | 0 | 3 | 6 |
| BMI < 20 | S. Salizzoni. et al.^37^ | 2016 | 1904 | 81.7 | 39.8 | 21.1 | 7.3 | 9.2 | 4 | 2 | 2 | 8 |
| BMI < 20 | E. Koifman. et al.^39^ | 2015 | 567 | 83.5 | 73.0 | NR | NR | 9.7 | 3 | 2 | 2 | 7 |
| BMI < 20 | E. Koifman. et al.^43^ | 2016 | 491 | 83 | 50 | NR | NR | 8.7 | 4 | 2 | 1 | 7 |
| BMI < 20 | T. Pilgrim. et al.^42^ | 2012 | 389 | 82.2 | 42 | 24 | NR | 6.8 | 3 | 0 | 3 | 6 |
| BMI < 20 | M. Yamamoto. et al.^44^ | 2013 | 3072 | 82,8 | 50,1 | 22,00 | NR | NR | 4 | 2 | 2 | 8 |
| BMI < 20 | H. Hioki H. et al.^34^ | 2018 | 1613 | 85 | 29.6 | NR | NR | 6.66 | 3 | 2 | 0 | 5 |
| ADL deficiency | Dziewierz A. et al.^49^ | 2017 | 148 | 82 | 37.8 | 14.5 | NR | 6.2 | 3 | 2 | 1 | 6 |
| ADL deficiency | Schoenenberger AW. et al.^60^ | 2018 | 330 | 83.6 | 43.4 | 19.2 | NR | 6 | 3 | 2 | 3 | 8 |
| Gait speed | Dziewierz A. et al.^49^ | 2017 | 148 | 82 | 37.8 | 14.5 | NR | 6.2 | 2 | 2 | 3 | 7 |
| Gait speed | Kano S. et al.^59^ | 2017 | 1256 | NR | 28.7 | 17.6 | 5.1 | 7.9 | 3 | 0 | 1 | 4 |
| Gait speed | P. Kleczynski. et al.^58^ | 2017 | 101 | 81 | 59 | 14 | NR | 12 | 3 | 2 | 3 | 8 |

ADL: Activity of Daily Living, BMI: Body Mass Index, CLD: Chronic Lung Disease, eGFR: estimated Glomerular Filtration Rate, NOS: Newcastle-Ottowa Scale
